# Neuroprotective Effects of Human Mesenchymal Stem Cells and Platelet‐Derived Growth Factor on Human Retinal Ganglion Cells

**DOI:** 10.1002/stem.2722

**Published:** 2017-10-31

**Authors:** Andrew Osborne, Julie Sanderson, Keith R. Martin

**Affiliations:** ^1^ John van Geest Centre for Brain Repair, University of Cambridge Cambridge United Kingdom; ^2^ School of Pharmacy University of East Anglia Norwich United Kingdom; ^3^ Cambridge NIHR Biomedical Research Centre Cambridge United Kingdom; ^4^ Eye Department Addenbrooke's Hospital Cambridge United Kingdom; ^5^ Wellcome Trust‐Medical Research Council, Stem Cell Institute Cambridge United Kingdom

**Keywords:** Mesenchymal stem cells, Neuroprotection, Human, Retina, Degeneration

## Abstract

Optic neuropathies such as glaucoma occur when retinal ganglion cells (RGCs) in the eye are injured. Strong evidence suggests mesenchymal stem cells (MSCs) could be a potential therapy to protect RGCs; however, little is known regarding their effect on the human retina. We, therefore, investigated if human MSCs (hMSCs), or platelet‐derived growth factor (PDGF) as produced by hMSC, could delay RGC death in a human retinal explant model of optic nerve injury. Our results showed hMSCs and the secreted growth factor PDGF‐AB could substantially reduce human RGC loss and apoptosis following axotomy. The neuroprotective pathways AKT, ERK, and STAT3 were activated in the retina shortly after treatments with labeling seen in the RGC layer. A dose dependent protective effect of PDGF‐AB was observed in human retinal explants but protection was not as substantial as that achieved by culturing hMSCs on the retina surface which resulted in RGC cell counts similar to those immediately post dissection. These results demonstrate that hMSCs and PDGF have strong neuroprotective action on human RGCs and may offer a translatable, therapeutic strategy to reduce degenerative visual loss. Stem Cells *2018;36:65–78*

**Significance Statement**

Stem cell therapies that work in rodent models are frequently proposed as potential treatments for human disease. Testing of such strategies in human tissue is an important interim between preclinical and clinical studies. Results of this study show that human mesenchymal stem cells and platelet‐derived growth factor provide significant protection to cells of the human retina, supporting observations seen in rodent models of optic nerve injury. This work highlights that similar, although not identical, mechanisms are responsible for retinal protection and that future stem cell‐based treatments may have the potential to reduce visual deterioration.

## Introduction

There has been considerable recent interest in the potential use of stem cells to treat a variety of neurodegenerative diseases. We and others have previously shown that injection of mesenchymal stem cells (MSCs) into the eye can significantly delay retinal ganglion cell (RGC) death in models of glaucoma and optic nerve injury 1, 2, 3, 4, 5, 6. Using a rat ex vivo retinal explant system, we demonstrated that much of the neuroprotective effect of MSCs was mediated by platelet‐derived growth factor (PDGF), a family of growth factors not previously associated with RGC survival in glaucoma 7. We also showed PDGF could preserve RGC synaptic proteins in vivo 8 and minimize axonal loss within the optic nerve in experimentally induced ocular hypertension 7, supporting further investigation of PDGF treatment as a potential future glaucoma therapy.

However, to date, no such studies have examined the translatability of these experiments to the human retina. Species differences in pathogenic and therapeutic pathways are conceivable 9 and may contribute to the poor rate of successful translation of treatment strategies from animal models to human clinical trials 10, 11, 12. Although the preclinical assessment of human RGC neuroprotection has proven challenging (partly due to the limitations of available animal models as well as tissue availability and difficulties in the culture of adult human RGCs), ex vivo retinal explants can provide a useful model to study some aspects of retinal disease 13, 14, 15. Importantly, we have shown good correlation between the ability of therapies to protect RGCs in explants and in vivo models of experimental glaucoma 16.

In the current study, we aimed to determine if human MSCs (hMSCs) or PDGF could reduce RGC loss and apoptosis in the human retina. By investigating the downstream signaling pathways following treatments, we also set out to clarify the mechanisms involved in human RGC neuroprotection and the impact of pathway inhibition. Through understanding the role of hMSC and hMSC‐derived factors on human tissue, we hope to accelerate and support future therapies to prevent visual deterioration.

## Materials and Methods

### Human Retinal Explant Culture

Donor human eyes were obtained <24 hours *post‐mortem* from the East Anglian Eye Bank (Norfolk and Norwich University Hospital) with research being conducted under the tenets of the Declaration of Helsinki with ethical approval from the U.K. National Research Ethics Committee (REC 04/Q0102/57 and REC 11/EE/0112). All donated eyes were free of diagnosed retinal pathology and contained no evidence of ocular trauma or retinal injury. In total, 34 human *post‐mortem* eye globes from 17 donors aged 36 to 78 years (Supporting Information Fig. 1C) were used for this study.

Human retinal explants were excised and cultured through a combination of previously published methods 13, 14, 15. The anterior portion of each donor eye was removed and the intact retina detached from the retinal pigmented epithelium via cuts around the ciliary body and at the optic nerve head. A flat retinal preparation was created and the macula removed using a 4‐mm diameter dissecting trephine (Biomedical Research Instruments, MD). Six smaller 3‐mm circular explants were taken equidistant from the macula in regions of comparable RGC number 14, 15 (Supporting Information Fig. 1A, 1B). Explants were cultured, photoreceptor side down, on polytetrafluoroethylene membranes (EMD Millipore, Billerica, MA) in 300 μl Neurobasal‐A media containing 2% B27 supplement, 1% N2 supplement, l‐glutamine (0.8 mM), penicillin (100 U/ml) and streptomycin (100 mg/ml) (All from Invitrogen, Paisley, U.K.) at an air‐fluid interface in 12 well plates (Corning, NY).

The six explants from a single retina represent an experimental (*n*) of 1, with each explant receiving a separate treatment as illustrated at the top of figures. This ensures all groups are age and donor matched to limit variation. Sometimes only four of the six explants were used in an experiment where certain explants were not deemed of good enough quality to include in the study or were used for other, unrelated experiments. Explants were cultured for up to 7 days ex vivo (7DEV) at 35°C in a humidified atmosphere of 95% air/5% CO_2_. Medium was either unchanged throughout experiments or, where stated, half the medium was replenished daily.

Retinal explants were treated with recombinant human PDGF‐AA or ‐AB (#100‐13A and 100‐00AB, Peprotech, Rocky Hill, NJ), and/or inhibitors of molecular signaling pathways, added to the culture medium at the beginning of experiments. A selection of explants were cocultured with hMSCs, added as a bolus 5 µl droplet of 5,000 cells to the RGC layer shortly after dissection. Inhibitors of PDGF and its downstream signaling pathways included PDGF neutralizing antibody (35 mg/ml; EMD Millipore, Billerica, MA), the phosphatidylinositol 3 (PI3) kinase inhibitor LY294002 (75 µM; Cell Signaling Technology, Danvers, MA), the ERK inhibitor U0126 (50 µM; Promega, Southampton, U.K.) and the STAT3 inhibitor S3I‐201 (100 µM; Sigma‐Aldrich, Poole, U.K.).

### hMSCs Culture

hMSCs were purchased from Stem Cell Technologies and cultured in DMEM (1 g/l glucose; Invitrogen, Paisley, U.K.) containing 10% fetal bovine serum (Invitrogen, Paisley, U.K.), and 1% penicillin and streptomycin (Thermo Scientific, Loughborough, U.K.). Cells were expanded from the same stocks as we have used previously 7 whereby the vendor had shown a >90% expression of CD29, CD44, CD105, and CD166 and <1% expression of CD14, CD34, and CD45. hMSCs were split at 75%–90% confluence and used between passages 9 and 14. For transplantation studies, hMSCs expression of the mesenchymal markers CD105 and CD73 was verified (Supporting Information Fig. 1D) and the cells were resuspended in explant medium prior to culture on the RGC layer of the retina. For conditioned medium investigation, hMSCs were grown in T75 tissue culture flasks (Corning, NY) and transferred to serum free medium for 72 hours.

### Immunohistochemistry and TUNEL Analysis

Immunohistochemistry was used to quantify the survival of RGC layer cell populations as previously demonstrated 14, 15, 16 and to visualize the activation and location of the PDGF receptors and their downstream signaling pathways within the retina. Explants were fixed in 4% paraformaldehyde (24 hours), cryopreserved in a 30% sucrose solution in PBS at 4°C (24 hours) before 13 μm sections were collected using a Bright OTF 5000 cryostat (Bright Instruments, Huntingdon, U.K.). Antigen retrieval (incubation for 5 minutes at 80°C in 10 mM Sodium Citrate Buffer, 0.05% Tween 20, pH 6.0) or methanol permeabilization (submersion for 5 minutes in ice cold 100% methanol) were used to enhance epitope‐antibody binding for phosphorylated and total PDGF receptors, MSC markers and phosphorylated‐STAT3 antibodies prior to blocking. Transverse sections were simultaneously blocked and permeabilized by incubation in 5% normal goat serum in PBS with 0.3% Triton X‐100 for 1 hour at room temperature.

Sections were incubated in primary antibody (Supporting Information Table 1) diluted in blocking solution overnight at 4°C, before incubation with secondary antibody diluted in blocking solution for 2 hours at room temperature. Nuclei were counterstained with DAPI.

The DeadEnd Fluorometric TUNEL system (TUNEL) (G3250 Promega, Southampton, U.K.) was performed alongside NeuN immunohistochemistry to visualize apoptotic RGCs. TUNEL staining was carried out in between primary and secondary antibody steps according to manufacturer's instructions and as previously performed 15. Samples were then mounted with fluorSave reagent (Calbiochem/EMD Chemicals Inc., Gibbstown, NJ) prior to imaging.

DAPI^+^, NeuN^+^, and TUJ1^+^ cell densities within the RGC layer were quantified by a masked investigator in at least eight sections per explant using a ×20 objective and a Leica DM6000 epifluorescence microscope (Leica Microsystems, Wetzlar, Germany). NeuN^+^ cells which also stained positive for TUNEL were identified as apoptotic RGCs. Visualization of PDGFR activation and downstream signaling were assessed using a Leica SP5 confocal microscope (Leica Microsystems, Wetzlar, Germany) equipped with a ×40 oil objective using a ×1.5 digital zoom and 0.5–0.8 sequential scanning z‐step interval.

### Western Blotting

Protein extraction was performed by lysing explants in Mammalian Protein Extract Reagent M‐PER supplemented with Halt Phosphatase Inhibitor Cocktail, Protease Inhibitor Cocktail and 5 mM EDTA (All from Thermo Scientific, Loughborough, U.K.). Tissue was homogenized on ice for 20 minutes and then centrifuged at 13,000 rpm for 5 minutes to isolate the soluble cell extract. Protein concentration was determined using a bicinchoninic acid protein assay (Thermo Scientific, Loughborough, U.K.).

Ten micrograms of protein was loaded into 4%–12% precast gels and electro‐transferred to polyvinylidene difluoride (PVDF) membranes (Thermo Scientific, Loughborough, U.K.). Membranes were blocked in 5% dried skimmed milk in PBS with 0.2% Tween20 (Sigma‐Aldrich, Poole, U.K.) for 1 hour and incubated overnight with primary antibody (Supporting Information Table 1) in blocking solution at 4°C. Horseradish peroxidase (HRP) conjugated secondary antibodies (1:10,000, Vector Laboratories Ltd., Peterborough, U.K.) were used for 2 hours before signal detection using ECL Prime (Amersham, GE Healthcare, U.K.) and an Alliance Western blot imaging system (UVItec Ltd., Cambridge, U.K.).

### Proteome Profiler Arrays

hMSCs conditioned media were concentrated 10–15× using Amicon Ultra‐15 centrifugal filter units (EMD Millipore, Billerica, MA) prior to analysis using commercially available human angiogenesis proteome profiler arrays (R&D systems, Abingdon, U.K.) according to the manufacturer's instructions. Membranes were developed using ECL Prime and a UVItec imaging system with antibody spot integrated density quantified using ImageJ.

### Lactate Dehydrogenase Assay

The level of necrotic cell damage was determined by measuring the lactate dehydrogenase (LDH) activity in treated cell culture medium according to the manufacturer's instructions (Roche Molecular Biochemicals, Burgess Hill, U.K.).

### ELISA

Human PDGF‐AB ELISA arrays (Roche Molecular Biochemicals, Burgess Hill, U.K.) were used to measure PDGF degradation in experiments.

### Statistical Analysis

Data are presented as the mean ± standard error of the mean (s.e.m) for all bar charts and scatter plots, with (*n*) representing the number of completely independent experiments performed on separate retinas. Comparisons between two groups were made using two‐tailed unpaired Student's *t* tests. Comparisons between three or more groups were made with one‐way ANOVA with Dunnett's post hoc test (GraphPad Prism; Graph‐Pad Software Inc., La Jolla, Ca) to compare experimental groups to controls if *p* < .05.

## Results

### Protection of Human RGCs by PDGF and hMSC Treatments

**Figure 1:**
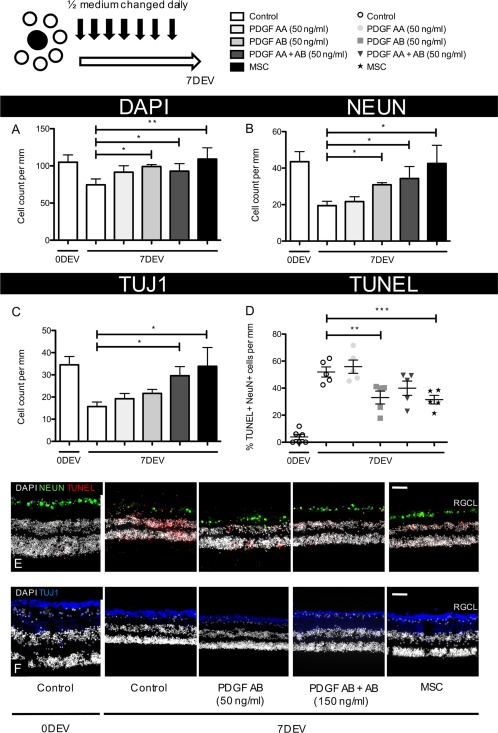
Human RGC neuroprotection by PDGF or human MSCs following regular medium replenishment. Human retinal explants were cultured for 7 days in medium containing PDGF or with the addition of hMSCs pipetted directly onto the RGC layer surface. **(A–C):** Neuronal survival in the RGC layer was quantified immediately post dissection, 0DEV or after 7 days’ culture (7DEV) (*n* = 6). **(D):** Number of apoptotic, NeuN^+^ cells were quantified and expressed as a percentage of all NeuN^+^ cells in the RGC layer (*n* = 6). **(E, F):** Representative images of RGCs (NeuN ‐ green or TUJ1 ‐ blue) in human retinal explants with apoptotic cells labeled red. ×20 objective, scale bar = 50 µm, *, *p* < .05; **, *p* < .01; ***, *p* < .001. Bar and scatter graphs show mean ± s.e.m with (*n*) number indicating the number of unique *post‐mortem* eyes used. The schematic in the top left shows the number of explants processed from each retina and the treatment time course. Abbreviations: 0DEV, 0 days ex vivo; 7DEV, 7 days ex vivo; MSC, mesenchymal stem cell; PDGF, platelet‐derived growth factor; RGCL, retinal ganglion cell layer.

Our previous work has demonstrated that MSCs can delay loss of axotomized RGCs in rodent retinal explants 16, with secreted PDGF playing a significant role in prolonging RGC survival 7. The current work expanded on those experiments by examining whether neuroprotection could also be demonstrated in a human retinal explant system. RGC number immediately post dissection, 0 days ex vivo (0DEV), provided an indication of the maximum number of surviving RGCs within explants. Over 7 days culture (7DEV) in control conditions, retinal thickness (Supporting Information Fig. 2A–2D) and the number of RGC layer cells decreased (Fig. 1A–1F). However, regular treatment of 50 ng/ml PDGF‐AB or ‐AA + AB could attenuate retinal thinning and RGC loss. Treatment of explants with PDGF‐AB increased the survival of both DAPI^+^ (99.2 ± 2.4 vs. 74.5 ± 7.2 cells/mm, *p* < .05, Fig. 1A, 1E) and NeuN^+^ neurons (30.9 ± 1.2 vs. 19.4 ± 2.4 cells/mm, *p* < .05, Fig. 1B, 1E) in the RGC layer compared with untreated explants (7DEV Control). Similarly, treatment with PDGF‐AA + AB protected RGC number (DAPI, 92.9 ± 9.3 vs. 74.5 ± 7.2 cells/mm, NeuN, 34.3 ± 6.6 vs. 19.4 ± 2.4 cells/mm, *p* < .05, Fig. 1A, 1B, 1E) compared with same day controls (7DEV Control). Quantification of TUJ1^+^ neurons in the RGC layer also demonstrated a comparable level of neuroprotection, although statistical significance was not reached with PDGF‐AB treatment (21.7 ± 1.8 vs. 15.7 ± 2.1 cells/mm, *p* = .070, Fig. 1C, 1F).

Even greater protection could be seen in explants cocultured with hMSCs compared with controls (DAPI, 109.2 ± 15.1 vs. 74.5 ± 7.2 cells/mm, NeuN, 42.6 ± 9.9 vs. 19.4 ± 2.4 cells/mm, TUJ1, 33.9 ± 8.4 vs. 15.7 ± 2.1 cells/mm, *p* < .05, Fig. 1A–1C, 1E, 1F) with a doubling in the number of surviving RGCs after 7 days and RGC counts that were similar to those measured immediately after dissection (0DEV Control).

Assessment of the apoptotic status of remaining RGC layer cells after 7 days revealed 33.0% ± 4.4% were apoptotic with PDGF‐AB treatment, 39.9% ± 4.8% with PDGF‐AA + AB and 31.5% ± 2.9% with hMSC coculture (Fig. 1D, 1E). Untreated explants at 7 days exhibited significantly greater levels of cell death with 51.9% ± 3.4% of remaining RGCs staining TUNEL positive. Of note, PDGF‐AA alone did not offer significant protection against cell loss nor apoptosis compared with untreated explants (Fig. 1A–1F).

### Effectiveness of a Single Treatment of PDGF‐AB on RGC Survival at 7 Days’ Ex Vivo

**Figure 2:**
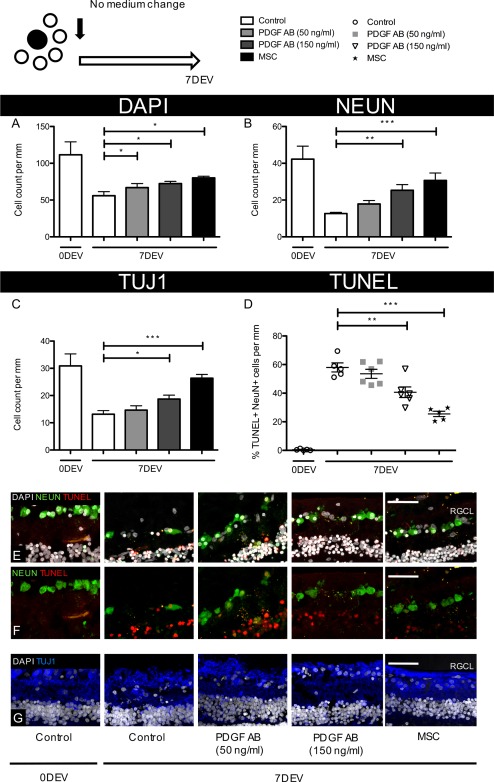
Human RGC neuroprotection after a single treatment of PDGF or human MSCs. Human retinal explants were cultured for 7 days in medium containing PDGF or with the addition of hMSCs pipetted directly onto the RGC layer surface. **(A–C):** Neuronal survival in the RGC layer was quantified immediately post dissection, 0DEV or after 7 days’ culture with no further medium change (7DEV) (*n* = 6) **(D):** Number of apoptotic, NeuN^+^ cells were quantified and expressed as a percentage of all NeuN^+^ cells in the RGC layer (*n* = 6). **(E, F):** Magnified, representative images of RGCs (NeuN ‐ green or TUJ1 ‐ blue) in human retinal explants with apoptotic cells labeled red. ×40 objective, scale bar = 50 µm, *, *p* < .05, **, *p* < .01 and ***, *p* < .001. Bar and scatter graphs show mean ± s.e.m with (*n*) number indicating the number of unique *post‐mortem* eyes used. The schematic in the top left shows the number of explants processed from each retina and the treatment time course. Abbreviations: 0DEV, 0 days ex vivo; 7DEV, 7 days ex vivo; MSC, mesenchymal stem cell; PDGF, platelet‐derived growth factor; RGCL, retinal ganglion cell layer.

We had previously not investigated ex vivo whether a single treatment with PDGF, as would be desirable in a human therapy, could mitigate RGC loss. A single supplement of 50 ng/ml PDGF‐AB added at day 0 offered minimal protection over the 7‐day time period, with marginally increased cell numbers compared with same time point controls (DAPI, 66.9 ± 5.5 vs. 60.0 ± 5.4 cells/mm, NeuN, 17.9 ± 1.9 vs. 12.7 ± 0.6 cells/mm, TUJ1 14.7 ± 1.6 vs. 13.1 ± 1.3 cells/mm, Fig. 2A–2C, 2E, 2G). Apoptosis was also slightly reduced but did not reach significance (7DEV PDGF‐AB (50 ng/ml), 53.5% ± 3.1% vs. 7DEV Control, 58.0% ± 2.8%, *p* = .348, Fig. 2D–2F). However, a higher concentration of PDGF‐AB (150 ng/ml) increased RGC layer survival (DAPI, 72.4 ± 3.0 vs. 60.0 ± 5.4 cells/mm, NeuN, 25.3 ± 3.1 vs. 12.7 ± 0.6 cells/mm, TUJ1, 18.7 ± 1.4 vs. 13.1 ± 1.3 cells/mm, *p* < .05, Fig. 2A–2C, 2E) and limited neuronal death (7DEV PDGF‐AB [150 ng/ml], 40.6% ± 3.7% vs. 7DEV Control, 58.0% ± 2.9%, *p* < .01, Fig. 2A–2G). Again, coculturing hMSCs on top of explants, even in the absence of medium changes, offered the greatest protection to RGCs in terms of both cell number and reducing apoptosis (Fig. 2A–2G).

**Figure 3:**
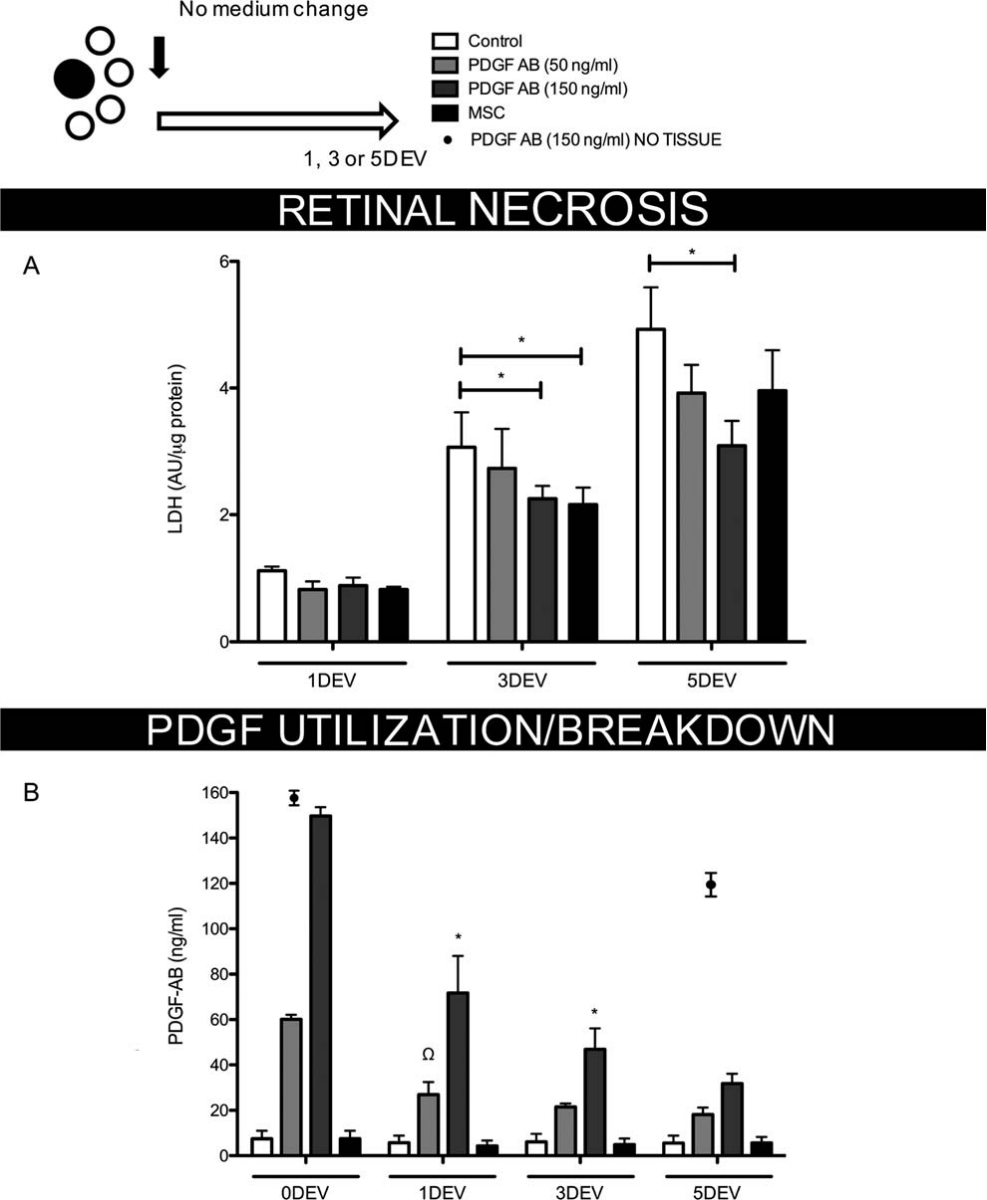
Necrotic cell damage in human retinal explants over time and the detectable levels of PDGF in the culture medium. **(A):** Lactate dehydrogenase within the culture medium relative to the protein content within human retinal explants treated with PDGF or hMSCs (*n* = 6), *, *p* < .05. **(B):** Detection of PDGF‐AB in the culture medium post addition at 0DEV (*n* = 6), *, *p* < .05 compared with 0DEV PDGF‐AB (150 ng/ml), ^Ω^ *p* < .05 compared with 0DEV PDGF‐AB (50 ng/ml). Bar graphs show mean ± s.e.m with (*n*) number indicating the number of unique *post‐mortem* eyes used. The schematic in the top left shows the number of explants processed from each retina and the treatment time course. Abbreviations: DEV, days ex vivo; MSC, mesenchymal stem cell; PDGF, platelet‐derived growth factor.

Sampling of the unchanged culture medium also revealed a steady increase in retinal tissue necrosis over time for all treatments. The rate of necrotic cell death was greatest in culture medium taken from untreated explants (Control, 0.95 ± 0.13 necrotic units per day) with reduced necrosis noted from 150 ng/ml PDGF‐AB or hMSC‐treated explants (respective 0.55 ± 0.32 and 0.78 ± 0.18 necrotic units per day, *p* < .05, Fig. 3A).

Assessment of PDGF‐AB concentration in the bathing medium at 1, 3, and 5 days provided a measure of PDGF utilization/degradation by retinal tissue. Initial sampling confirmed the correct concentration of PDGF‐AB had been administered to explants and levels approximately halved after 1 day in culture (0DEV PDGF‐AB [150 ng/ml], 149 ± 3.8 vs. 1DEV PDGF‐AB [150 ng/ml] 71.69 ± 16.4 ng/ml; 0DEV PDGF‐AB [50 ng/ml], 60.1 ± 2.0 vs. 1DEV PDGF‐AB [50 ng/ml] 26.9 ± 5.5 ng/ml, Fig. 3B). By 5 days, PDGF concentration within culture medium had decreased to 31.8 ± 4.3 ng/ml in the 150 ng/ml PDGF‐AB treatment group, a decrease of 78.8% from initial levels. Interestingly, PDGF‐AB could not be detected in culture medium taken from hMSCs treated explants (Fig. 3B) although PDGF‐AA and AB were measurable in the secretome of hMSC when cultured independent of retinal tissue (Fig. 7G).

### Adverse Effects of PDGF‐AB and hMSC Treatments on the Retina

It is worth noting that the effects of PDGF and hMSCs were not limited to RGCs, with changes in both glial and microglia activity observed after treatments (Supporting Information Fig. 3). 7 days after a single dose of PDGF‐AB or coculture with hMSC, extensive gliosis and inflammation was detectable throughout the retina (Supporting Information Fig. 3A) with proliferating macrophages seen within the inner retina (Supporting Information Fig. 3B). Western blotting confirmed GFAP and IBA1 immunoreactivity were significantly elevated in retinal explants treated with PDGF‐AB or hMSC compared with same time point controls (GFAP, PDGF‐AB [150 ng/ml] 2.5 ± 0.3, hMSC 3.0 ± 0.2‐fold; IBA1, PDGF‐AB [150 ng/ml] 1.3 ± 0.1, hMSC 1.7 ± 0.2‐fold increase at day 5, *p* < .05, Supporting Information Fig. 3C, 3D).

### Downstream Signaling Pathway Activation Within the Retina with PDGF‐AB or hMSCs Treatments

**Figure 4:**
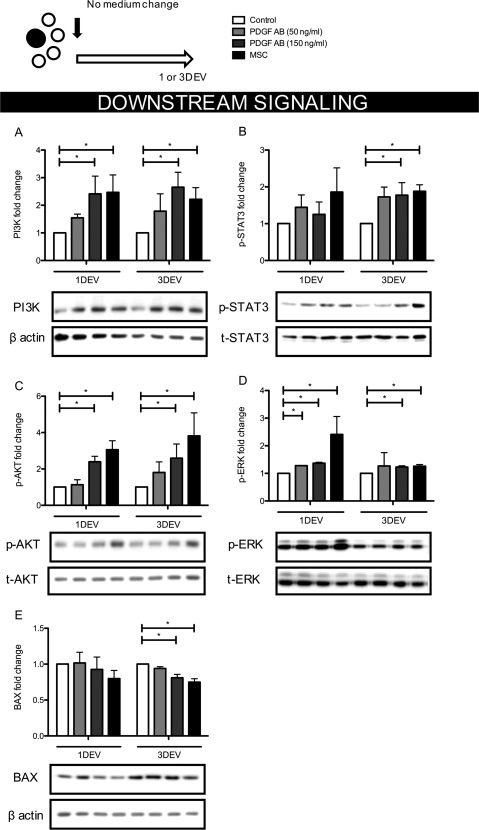
Activation of downstream signaling pathways in human retinal explants after a single treatment with PDGF or human MSCs. **(A–D):** Activation of cell survival pathways 1 and 3 days after human explant culture in PDGF supplemented medium or addition of hMSCs relative to untreated, same time point controls (*n* = 4), *, *p* < .05. **(E):** Pro‐apoptotic BAX expression in treatment groups relative to untreated, same time point controls (*n* = 4), *, *p* < .05. Bar graphs show mean ± s.e.m with (*n*) number indicating the number of unique *post‐mortem* eyes used. The schematic in the top left shows the number of explants processed from each retina and the treatment time course. Abbreviations: DEV, days ex vivo; MSC, mesenchymal stem cell; PDGF, platelet‐derived growth factor.

Having shown both PDGF‐AB and hMSCs offered significant neuroprotection to human RGCs, we investigated the activation of downstream signaling pathways shortly after each treatment. Downstream signaling of survival pathways PI3K, AKT, STAT3, and ERK were upregulated after 150 ng/ml PDGF‐AB or hMSC treatment versus controls at both 1 and 3 days (PI3K 2.7 ± 0.5 and 2.2 ± 0.4, AKT 2.6 ± 0.8 and 3.8 ± 1.3, STAT3 1.9 ± 0.15 and 1.8 ± 0.3, ERK 1.2 ± 0.1 and 1.3 ± 0.1‐fold increase at day 3, *p* < .05, Fig. 4A–4D) which correlated with reduced retinal pro‐apoptotic BAX expression (down 18.9% ± 3.0% at 3 days compared with 3DEV Control, *p* < .05, Fig. 4E). Lower concentrations of PDGF‐AB showed reduced signaling pathway activation that did not reach levels significantly different from controls (PI3K 1.8 ± 0.6, AKT 1.8 ± 0.5, STAT3 1.7 ± 0.2, ERK 1.3 ± 0.4‐fold increase at day 3, Fig. 4A–4D). Consistent with this lack of activation was no clear reduction in BAX expression (3DEV PDGF‐AB [50 ng/ml] down 6.0% ± 3.2% vs. 3DEV Control, *p* = .074, Fig. 4E).

**Figure 5:**
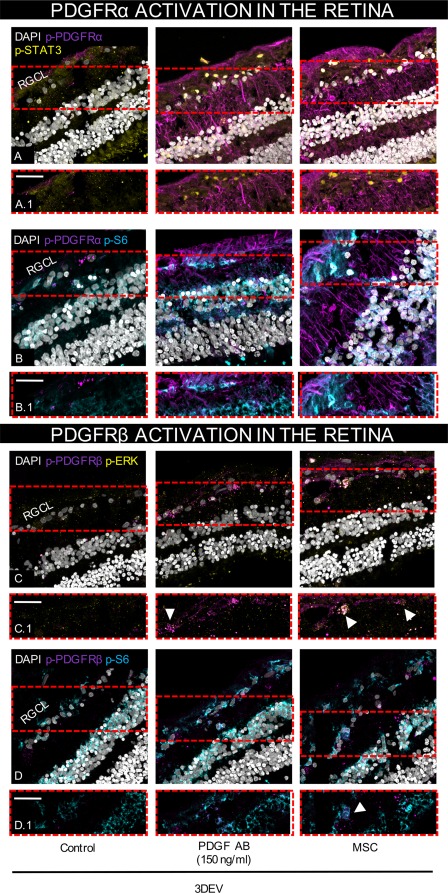
PDGFR activation and downstream survival signaling could be seen within the retina and RGCL after PDGF and human MSC treatments. **(A):** Increased PDGFRα signaling (purple) was observed 3 days after PDGF‐AB or hMSC treatments with elevation in phosphorylated STAT3 (p‐STAT3 ‐ yellow) and phosphorylated S6 (p‐S6 ‐ teal) signaling in the RGCL. PDGFRβ activation (purple) was detected on RGCL cells and colocalized with activated ERK (p‐ERK ‐ yellow) or p‐S6 (teal). ×40 objective, scale bar = 50 µm, arrows show colocalization in the RGCL. Representative images from three experimental repeats. Abbreviations: DEV, days ex vivo; MSC, mesenchymal stem cell; PDGFR, platelet‐derived growth factor receptor; RGCL, retinal ganglion cell layer.

Immunohistochemistry for the phosphorylated PDGF receptors α and β (PDGFRα and β), including downstream signaling activation, revealed similar labeling within the retina after both PDGF‐AB and hMSC treatments. Minimal receptor phosphorylation was observed in untreated explants with little detectable downstream signaling pathway activation (Fig. 5A–5D). In both PDGF and hMSC groups, phosphorylated PDGFRα was detected within the RGCL and inner nuclear layer with labeling likely along Müller glia processes (Fig. 5A, 5B). Activated PDGFRβ expression was restricted to the RGC layer with colocalization observed with the downstream PI3K ribosomal protein S6 kinase and phosphorylated ERK (Fig. 5C, 5D). Activated PDGFRβ could be seen in TUJ1^+^ cells indicating a direct effect of treatments on RGCs (Supporting Information Fig. 4A). PDGFRα activation showed no clear colocalization with either S6 or phosphorylated STAT3 which were both activated on RGCL cells after PDGF or hMSC treatment (Fig. 5A, 5B). Total PDGF receptor expression did not differ with treatment, with total PDGFRβ expressed on RGC layer cells and the occasional blood vessel within the retina. Total PDGFRα was expressed throughout the retinal layers (Supporting Information Fig. 4B, 4C).

### Effects of Inhibiting PDGF Signaling on Retinal and RGC Apoptosis

**Figure 6:**
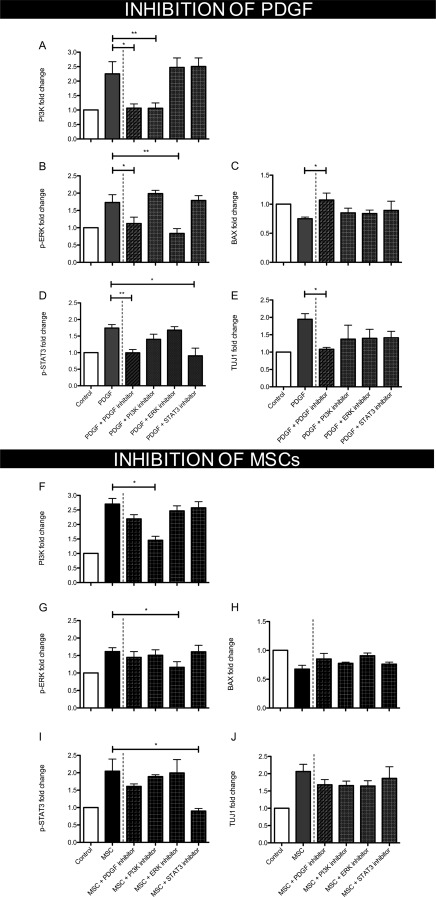
The beneficial effects of PDGF or human MSCs could be reduced using a variety of inhibitors when assessed 3 days after treatment. **(A–E):** PDGF in combination with a PDGF inhibitor no longer activated survival pathways or protected against a decrease in retinal ganglion cell (TUJ1) expression (*n* = 4). Inhibitors of PI3K, ERK, or STAT3 effectively blocked individual pathway signaling but did not improve retinal (BAX) or retinal ganglion cell specific (TUJ1) survival (*n* = 4). **(F–J):** hMSCs could still offer some protection even in the presence of inhibitors against PDGF, PI3K, ERK, or STAT3 (*n* = 4), *, *p* < .05; **, *p* < .01. Bar graphs show mean ± s.e.m with (*n*) number indicating the number of unique *post‐mortem* eyes used. Abbreviations: MSC, mesenchymal stem cell; PDGF, platelet‐derived growth factor.

The impact of using a PDGF neutralizing antibody in both PDGF and hMSC‐treated explant experiments was assessed to better understand the implications for downstream signaling and their possible effect on retinal cell death. In PDGF‐AB treated explants, anti‐PDGF treatment successfully prevented PI3K, STAT3, and ERK signaling and removed any protective effects on BAX expression (Fig. 6A–6D). TUJ1 expression, indicative of RGC survival, was also significantly decreased when using the neutralizing antibody (from a 1.9 ± 1.5‐fold increase from control using PDGF to a 1.1 ± 0.4‐fold increase from control when adding the PDGF inhibitor, *p* < .05, Fig. 6E). Selective inhibition of each signaling pathway using commercially available inhibitors resulted in a slight increase in apoptotic BAX expression and a modest decrease in TUJ1 expression, however less evident than with the PDGF neutralizing antibody (Fig. 6C, 6E).

**Figure 7:**
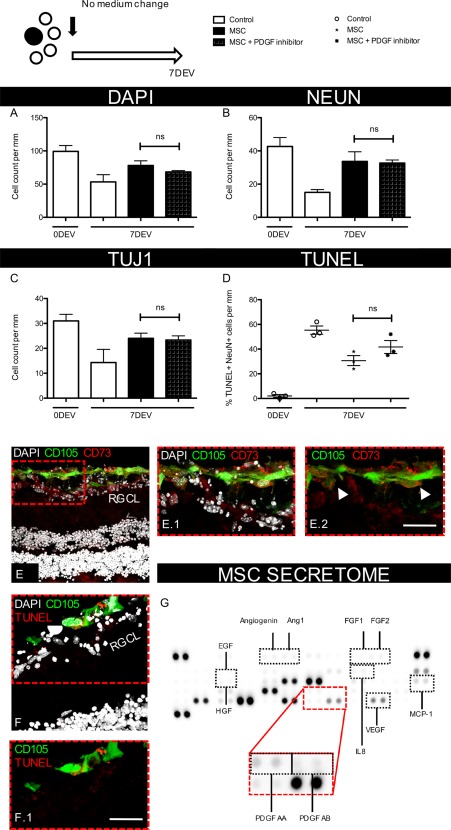
Inhibition of PDGF in human retinal explants cultured with human MSCs did not prevent MSC mediated neuroprotection. **(A–C):** Neuronal survival in the RGCL was quantified immediately post dissection, 0DEV or after 7 days’ culture with no further medium change (7DEV) (*n* = 3). **(D):** Number of apoptotic, NeuN^+^ cells were quantified and expressed as a percentage of all NeuN^+^ cells in the RGC layer (*n* = 3). **(E):** Human MSCs (CD105^+^ ‐ green and CD73^+^ ‐ red cells) could be visualized on the surface of the human retina in close proximity to the RGC layer. **(F):** Human MSCs remained viable throughout the 7‐day culture with little evidence of apoptotic labeling (red staining on green cells). **(G):** Multiple growth factors and neurotrophins could be measured in the hMSC secretome after 72 hours in culture. ×40 objective, scale bar = 50 µm. Bar and scatter graphs show mean ± s.e.m with (*n*) number indicating the number of unique *post‐mortem* eyes used. The schematic in the top left shows the number of explants processed from each retina and the treatment time course. Abbreviations: 0DEV, 0 days’ ex vivo; 7DEV, 7 days ex vivo; Ang, angiopoietin; EGF, epidermal growth factor; FGF, fibroblast growth factor; HGF, hepatocyte growth factor; IL, interleukin; MSC, mesenchymal stem cell; MCP, monocyte chemotactic protein; PDGF, platelet‐derived growth factor; RGCL, retinal ganglion cell layer; VEGF, vascular endothelial growth factor.

In hMSC experiments, addition of the PDGF neutralizing antibody to explants reduced but did not significantly inhibit PI3K, STAT3 and ERK signaling (Fig. 6F–6I), indicating other secreted neurotrophin could also be stimulating these pathways. Some of these neurotrophin were detected in hMSC culture medium, many of which were at higher concentrations than PDGF (Fig. 7G). BAX and TUJ1 expression were not significantly changed with the PDGF neutralizing antibody or individual pathway inhibition (Fig. 6H–6J). Supporting the observation that inhibiting PDGF did not prevent hMSC induced neuroprotection, RGC cells were quantified in explants cocultured with hMSCs plus or minus the PDGF neutralizing antibody (Fig. 7). Blocking PDGF in these experiments had no significant impact on RGC survival with similar cell counts between hMSC and hMSC + PDGF inhibitor groups (DAPI, 78.1 ± 7.2 vs. 68.3 ± 2.0 cells/mm, NeuN, 33.7 ± 5.8 vs. 32.4 ± 1.9 cells/mm, TUJ1 24.1 ± 2.2 vs. 23.2 ± 1.7 cells/mm, Fig. 7A–7C). Also, no significant differences were identified between the number of apoptotic RGCs (7DEV hMSC, 30.7% ± 4.1% vs. 7DEV hMSC + PDGF inhibitor, 41.5% ± 5.2%, *p* = .172, Fig. 7D) supporting the assumption that PDGF is not essential for hMSC mediated neuroprotection.

To ensure hMSCs remained attached to retinal explants throughout the 7‐day culture period, a selection of retinal sections was stained with mesenchymal markers CD105 and CD73 (Fig. 7E) which were also examined in culture prior to transplantation (Supporting Information Fig. 1D). Both markers detected a layer of cells on the surface of the retina positive of mesenchymal lineage which remained viable over the time courses investigated with little apoptotic TUNEL staining colocalized with CD105^+^ (Fig. 7F).

## Discussion

Clinical trials into the effectiveness of MSC‐based therapies are currently underway for neuronal and 17 non‐neuronal diseases 18. As yet, no MSC‐based treatments have reached Phase II clinical trials for glaucoma (clinicaltrials.gov) 19, despite promising results in animal models.

The current work provides evidence that hMSC‐based treatments can be neuroprotective to the human retina. These results complement previous rodent observations that both hMSC and PDGF‐AB offer robust protection in retinal explant systems modeling axonal injury, and could offer attractive therapeutic strategies. Intriguingly, in human retinas, we showed that PDGF‐AA treatment was ineffective at delaying RGC loss or apoptosis, a difference to previous observation in rats 7. The isoform PDGF‐AB however was strongly neuroprotective and showed a dose dependent response when administered as a single treatment shortly after RGC axotomy.

Regarding the discrepancy in terms PDGF isoform protection between species, it is possible that differences in the expression pattern of PDGF receptors on rodent and human RGCs are responsible. PDGF ligands bind two structurally related tyrosine kinase cell surface receptors, α and β which initiate signal induction 20, 21. PDGF‐AA exclusively binds PDGFRα whereas PDGF‐AB facilitates both PDGFRα and β dimerization and subsequent phosphorylation.

In rats, PDGFRα phosphorylation after PDGF‐AB treatment can be detected in RGCs and Müller glia 7, 8 implying direct survival pathway activation occurs within the RGC layer. In mice, PDGFRα phosphorylation after PDGF‐AB treatment is also upregulated throughout the retina 22; however, human PDGFRα phosphorylation is absent from RGCs. Only activated PDGFRβ was detected on human RGC signifying that activation of the β receptor may be necessary for RGC protection, which supports the protective effects seen with PDGF‐AB. A similar pattern of PDGFRβ activation on RGCs has been observed in mice shortly after PDGF‐AB treatment, although a direct correlation between β activation and downstream signaling was not explored 22.

Of the receptor isoforms, there is strong evidence linking PDGFRβ activation to enhanced neuronal survival after focal ischemia 23, 24 and NMDA‐induced cell death 25, 26, 27. The PDGFRβ has also been shown to be involved in reducing the progressive loss of tyrosine hydroxylase‐positive neurons in rat and human cell cultures, with PDGF‐BB but not PDGF‐AA reducing neuronal damage 28. Interestingly, PDGF‐BB treated cultures of human but not rat neurons had longer neurites than control or PDGF‐AA treated cultures 28, supporting our observations that differences exist between species. Furthermore, knock out of PDGFRβ in neurons increases their susceptible to excitotoxic cell death 29.

PDGF‐BB has also been shown to have comparable neuroprotective effects to brain‐derived neurotrophic factor in human tyrosine hydroxylase‐positive neurons 30 and is being considered a clinical candidate drug for treatment of Parkinson's disease after promising results in relation to restoring dopaminergic neurotransmission and functional recovery in vivo 31, 32. This would be in keeping with our data suggesting PDGFRβ activation may provide a therapeutic target for RGC neuroprotection. Further work using PDGF‐BB, which binds PDGFRβ and not PDGFRα 33, would be useful to confirm the importance of each receptor in the human retina.

In addition to the beneficial effect of PDGF‐AB, we have shown for the first time that hMSCs can protect human RGC ex vivo and mediated even stronger protection in terms of delaying cell loss and minimizing apoptosis. After 7 days in culture, hMSCs could completely prevent RGC loss, strengthening the appeal of MSC‐based therapy for optic nerve diseases such as glaucoma in the future. It is widely acknowledged that MSCs do not integrate into the retina 6, 34 and, therefore, the observed protection relates to secreted trophic support rather than cell replacement.

Although we could detect PDGFRα and β activation following hMSC treatment, measured levels of secreted PDGF in the bathing medium were low. However, assessment of the MSC secretome in the absence of explants, via proteome profiler analysis, did reveal both PDGF‐AA and AB were secreted by hMSC. Previous analysis of the hMSC secretome has also indicated that after 3 days in culture, secreted PDGF‐AB is within the pg/ml concentration range 7, below the detection limits of our ELISA. It is predicted that the concentration of neurotrophins could be several orders of magnitude higher at inner retinal surface if MSC and RGCs were in close proximity. This could explain our observation of similar levels of PDGFR activation after PDGF‐AB or hMSC treatment despite the considerable differences in quantifiable PDGF within explant bathing medium. Even so, our experiments investigating the inhibition of PDGF in human retinal explants treated with hMSC suggests other neurotrophins are likely to have a beneficial effect on the retina and the effects of PDGF are not exhaustive.

With regard to mechanisms of survival, many RGC neuroprotective and regenerative strategies focus on the upregulation of MTOR signaling, which is often diminished with injury 35, 36, 37. The mTOR signaling pathway has a pivotal role in numerous cellular processes and can be upregulated through elevated PI3K/AKT and MAPK/ERK signaling 37. Activation of mTOR activation leads to the phosphorylation of S6 and STAT3 which have been correlated with improved RGC neuronal survival, cell growth, protein synthesis and delayed apoptosis 38, 39, 40, 41, 42. For this reason, many RGC‐based strategies have focused on targeting one or more of these pathways with promising outcomes on protection in models of optic nerve injury and glaucoma 38, 43, 44, 45.

Our data showed that both PDGF and hMSC treatments increased numerous neuroprotective pathways and the phosphorylation of AKT, ERK, and STAT3 inversely correlated to apoptotic protein expression as has been observed by others 46, 47. It was noted that discrete pathway inhibition could not accelerate human retinal cell apoptosis implying multiple pathways contribute to RGC survival after injury.

Interestingly, although the extent of downstream signaling pathway activation was comparable between PDGF and hMSC‐treated explants, overall RGC survival was greater within the hMSC treatment group. The enhanced protection with hMSCs is likely due to the large number of other neurotrophins being secreted 7, 48, 49 which could bind a larger number of receptors present on RGCs. It was noticed that PDGFRβ was not present on all RGCs and therefore protection could be limited to specific subtypes of RGC. Therefore, MSC therapy may provide a more reliable treatment for diseases of complex pathophysiology like glaucoma 50.

Potential hurdles do however exist with regard to the use of MSCs for retinal diseases. First, it is challenging to control the factors secreted by MSCs and how these factors could change over time and with disease progression. Second, questions remain regarding the tumorigenic potential of such cells. Third, MSCs also secrete factors that cause extensive reactive gliosis 34, 51 and inflammation which could contribute to retinal detachment 51, 52. In human explants, we observed increased microglia proliferation and gliosis after hMSC treatment, similar to observations in our rodent animals models 34, 51. This would have serious consequences to a patient's vision and presents a barrier to MSC‐based treatment without suitable anti‐inflammatory strategies. We originally hypothesized that the selective use of a single growth factor would circumvent some of these adverse effects, but PDGF led to similar inflammation and gliosis in the human retina, as recently observed in rodents following PDGF therapy 8.

## Conclusion

This study demonstrates that both hMSCs and PDGF have strong neuroprotective actions and may be useful as part of a strategy to prevent degenerative visual loss in the future if adverse effects on retinal glia can be mitigated.

## Author Contributions

A.O.: conception and design, financial support, collection and/or assembly of data, data analysis and interpretation, manuscript writing; J.S.: administrative support, provision of study material or patients, final approval of manuscript; K.R.M.: conception and design, data analysis and interpretation, financial support, administrative support, final approval of manuscript.

## Disclosure of Potential Conflicts of Interest

The authors indicated no potential conflicts of interest.

## Note Added in Proof

This article was published online on 31 October 2017. Minor edits have been made that do not affect data. This notice is included in the online and print versions to indicate that both have been corrected 29 December 2017.

## Supporting information

Supporting Information Figure 1

Supporting Information Figure 2

Supporting Information Figure 3

Supporting Information Figure 4

Supporting Information Table 1
