# Supplementary figures and images for: Neuroprotective Effects of Human Mesenchymal Stem Cells and Platelet‐Derived Growth Factor on Human Retinal Ganglion Cells
Source: Stem Cells. 2017 Oct 31;36(1):65–78. doi: 10.1002/stem.2722 (PMC5765520; doi:10.1002/stem.2722)

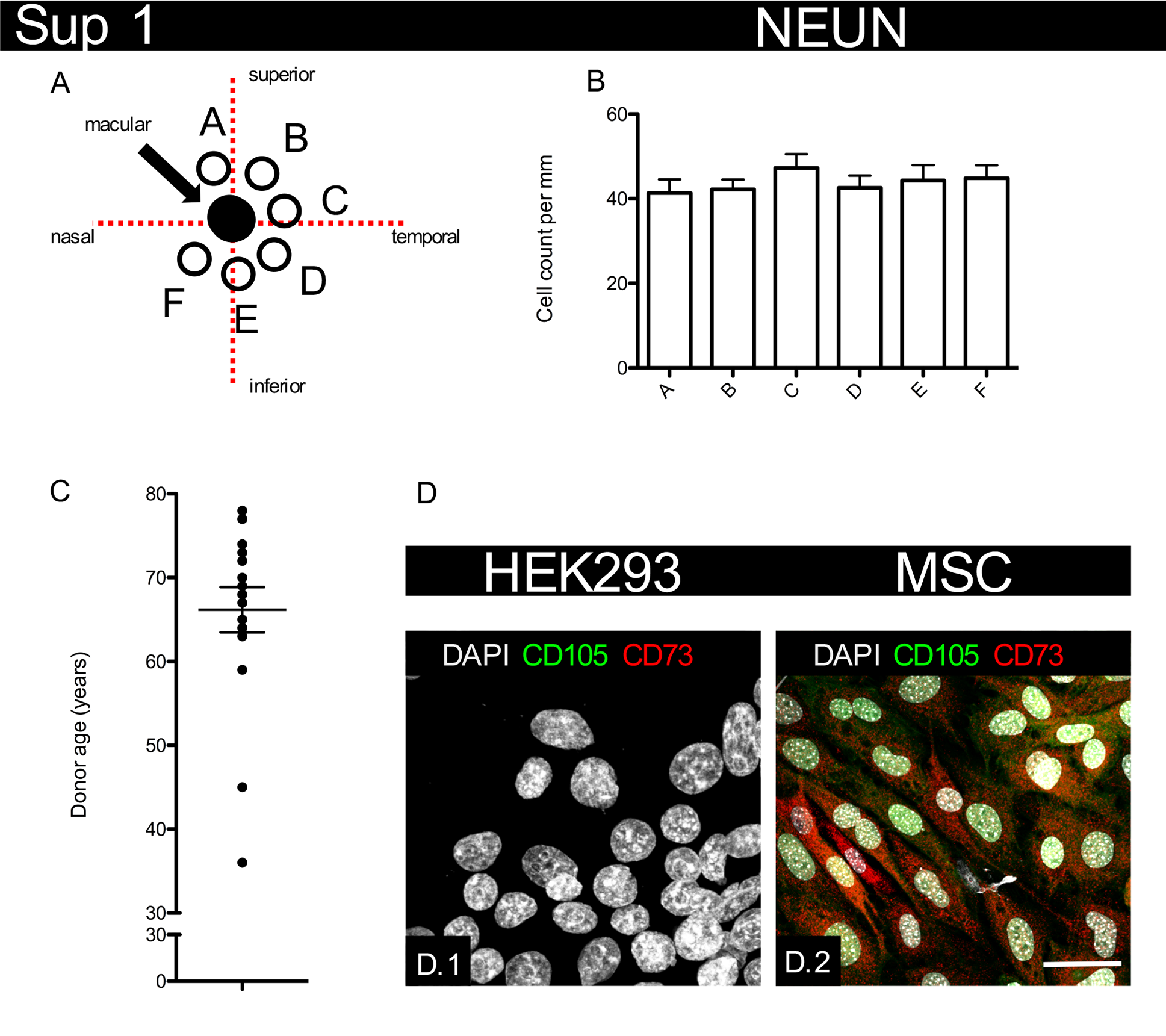

Supplement: Supplementary file 1 — Supporting Information Figure 1 [file STEM-36-65-s001.tif]

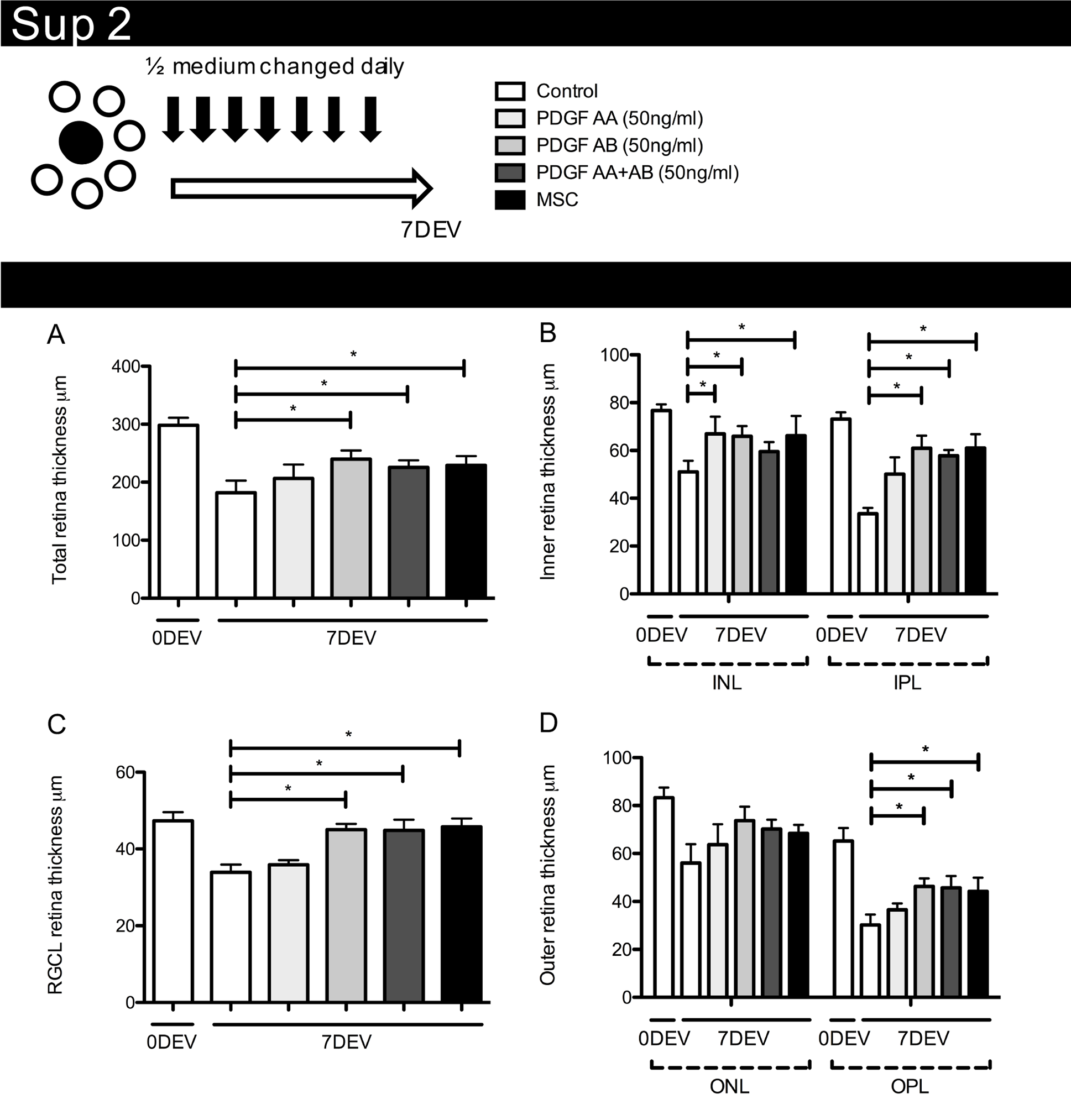

Supplement: Supplementary file 2 — Supporting Information Figure 2 [file STEM-36-65-s002.tif]

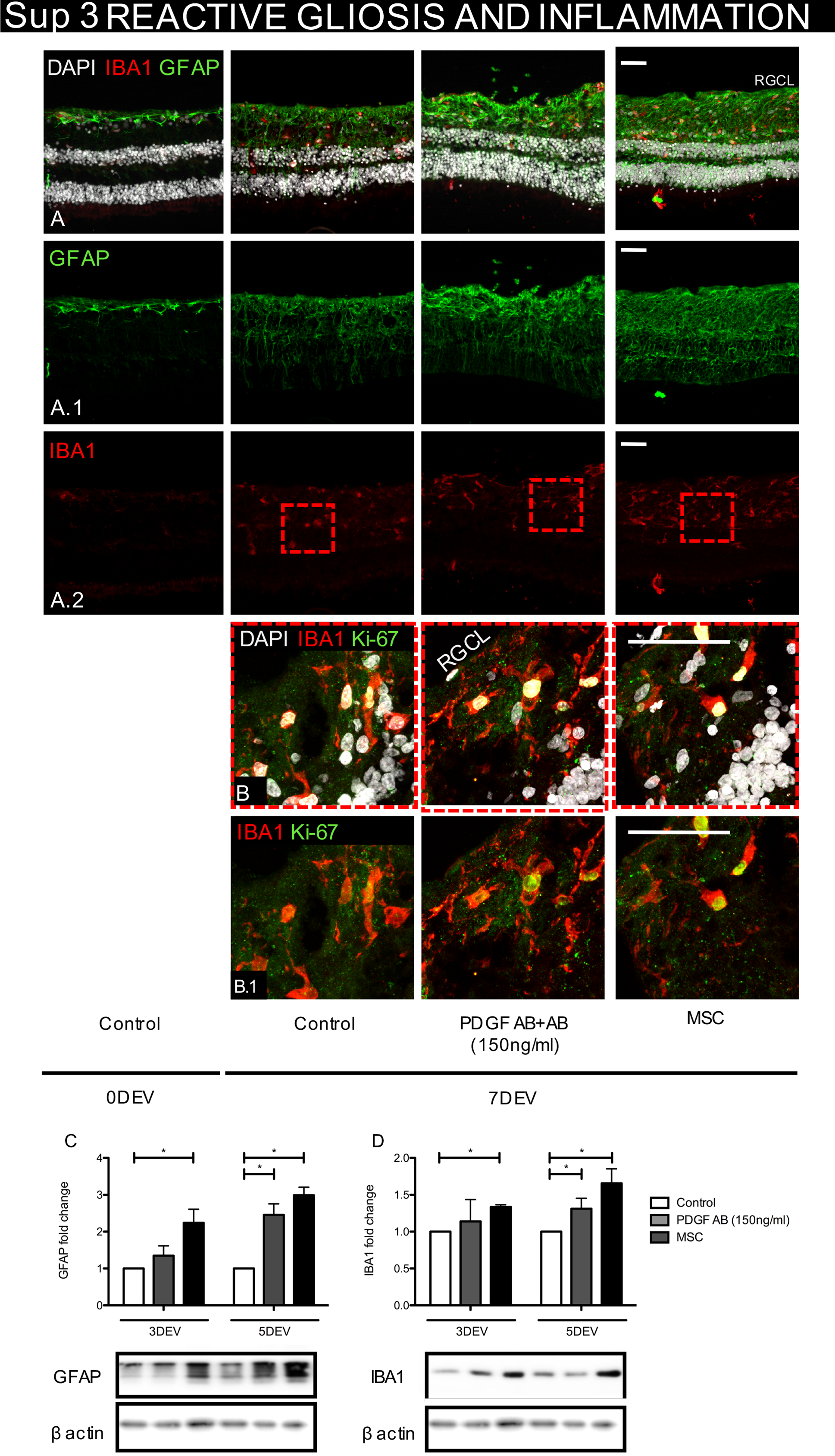

Supplement: Supplementary file 3 — Supporting Information Figure 3 [file STEM-36-65-s003.tif]

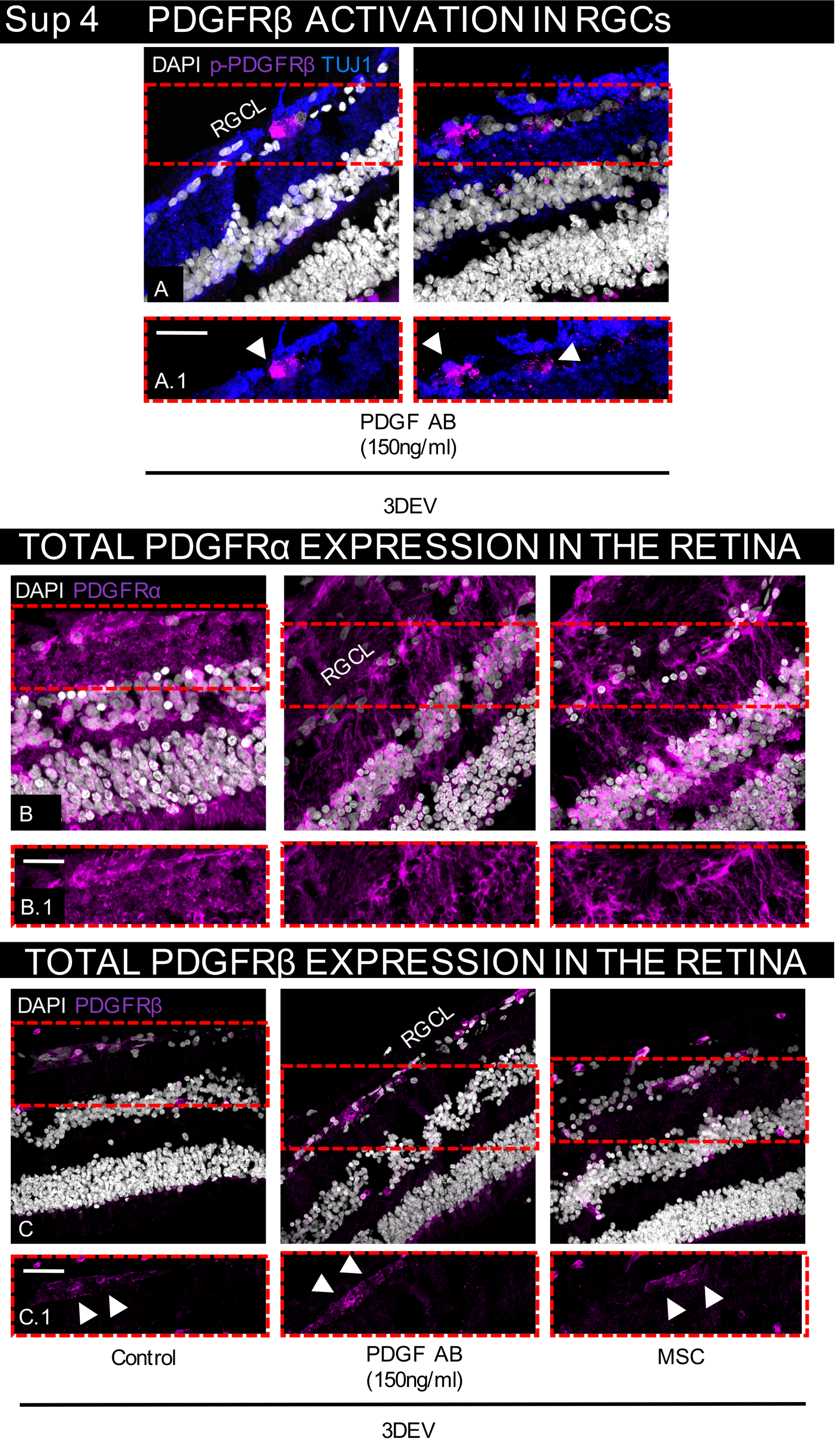

Supplement: Supplementary file 4 — Supporting Information Figure 4 [file STEM-36-65-s004.tif]
