# Supplementary material for: Neuroprotective Effects of Human Mesenchymal Stem Cells and Platelet‐Derived Growth Factor on Human Retinal Ganglion Cells
Source: Stem Cells. 2017 Oct 31;36(1):65–78. doi: 10.1002/stem.2722 (PMC5765520; doi:10.1002/stem.2722)
Supplement: Supplementary file 5 — Supporting Information Table 1 [file STEM-36-65-s005.docx]

Table 1: Primary antibodies

| Specificity | Source | Isotype | Company | Concentration |
| --- | --- | --- | --- | --- |
| NeuN | Mouse | Monoclonal | Millipore | 1:250 (IHC) |
| TUJ1 | Mouse | Monoclonal | Promega | 1:400 (IHC)  1:500 (WB) |
| PI3K | Rabbit | Monoclonal | Cell Signaling | 1:750 (WB) |
| p-STAT3  (Tyr705) | Rabbit | Monoclonal | Cell Signaling | 1:1000 (WB) |
| p-STAT3  (Tyr705) | Mouse | Monoclonal | Cell Signaling | 1: 200 (IHC) |
| t-STAT3 | Rabbit | Monoclonal | Cell Signaling | 1:1000 (WB) |
| p-AKT (Ser473) | Rabbit | Polyclonal | Cell Signaling | 1:500 (WB) |
| t-AKT | Rabbit | Polyclonal | Cell Signaling | 1:1000 (WB) |
| p-ERK1/2 (Thr202/Tyr204) | Rabbit | Monoclonal | Cell Signaling | 1:500 (WB) |
| p-ERK1/2 (Thr202/Tyr204) | Mouse | Monoclonal | Cell Signaling | 1:300 (IHC) |
| ERK1/2 | Rabbit | Monoclonal | Cell Signaling | 1:1000 (WB) |
| BAX | Rabbit | Polyclonal | Cell Signaling | 1:500 (WB) |
| β-actin | Rabbit | Polyclonal | Cell Signaling | 1:1000 (WB) |
| p-PDGFRα | Mouse | Monoclonal | Abcam | 1:50 (IHC) |
| p-PDGFRβ | Mouse | Monoclonal | Cell Signaling | 1:100 (IHC) |
| p-PDGFRβ | Rabbit | Monoclonal | Cell Signaling | 1:100 (IHC) |
| t-PDGFRα | Rabbit | Monoclonal | Abcam | 1:200 (IHC) |
| t-PDGFRβ | Rabbit | Monoclonal | Abcam | 1:100 (IHC) |
| p-S6 (Ser235/236) | Rabbit | Polyclonal | Cell Signaling | 1:250 (IHC) |
| p-S6 (Ser235/236) | Mouse | Monoclonal | Santa Cruz | 1:200 (IHC) |
| CD105 | Mouse | Monoclonal | Abcam | prediluted (IHC) |
| CD73 | Rabbit | Polyclonal | Abcam | 1:200 (IHC) |
| IBA1 (AIF1) | Guinea Pig | Polyclonal | Synaptic Systems | 1:500 (IHC) |
| IBA1 (AIF1) | Rabbit | Polyclonal | WAKO | 1:500 (WB) |
| GFAP | Rabbit | Polyclonal | DAKO | 1:500 (IHC)  1:1000 (WB) |
| Ki-67 | Rabbit | Polyclonal | Abcam | 1:500 (IHC) |
